# Demographic Factors Driving Schistosomiasis and Soil-Transmitted Helminthiases in Milola Ward, Lindi District, Tanzania: A Useful Guide for Launching Intervention Programmes

**DOI:** 10.24248/EAHRJ-D-18-00008

**Published:** 2018-11-23

**Authors:** Jared Bakuza

**Affiliations:** a Department of Biological Sciences, Dar es Salaam University College of Education, Dar es Salaam, Tanzania

## Abstract

**Background:**

Current information on the distribution of and risk factors for schistosomiasis and soil-transmitted helminthiases is scarce for most areas of southern Tanzania, including Milola Ward in Lindi District. This study was initiated to establish the status of these infections in Milola Ward and to assess how they vary with demographic factors.

**Methods:**

From September to October 2014, 2 sets of stool and urine samples were collected from residents of Milola Ward. The Kato–Katz technique was used to examine stool samples for faecal-borne parasites, and the filtration technique was used to examine urine for urinary schistosomes. A total of 195 individuals aged 5 to 90 years were enrolled in the study; 190 (97%) participants submitted adequate urine samples, of whom 107 (56%) were female and 83 (43%) were male. Of the 195 participants who took part in the initial sampling exercise, 158 (81%) provided adequate stool samples; 121 (77%) of these were adults, and the rest (n=37, 23%) were children. Only 53 urine and 26 faecal samples were obtained in the second round of sampling, and due to marked inconsistencies, these have been excluded from the analysis. Mean parasite abundance was analysed for its association with demographic factors, such as age and sex.

**Results:**

Three varieties of parasite were detected, namely, *Schistosoma haematobium* in 44 (23%) of 190 urine samples, hookworms in 12 (8%) of 158 stool samples, and *Trichuris trichiura* in 6 (4%) of 158 stool samples. The difference in *S. haematobium* prevalence between male and female participants (27 of 107 females, 25% vs 17 of 83 males, 20%) was not statistically significant (Kruskal–Wallis test, *P*=.47). Linear regression analysis of *S. haematobium* infection with age showed a significant association, with children having higher infection intensities than adults (*P*<.001). *S. haematobium* prevalence and intensity did not vary significantly between villages (intensity [Kruskal–Wallis test], *P*=.95; prevalence, *P*=.88).

**Discussion:**

These data confirm that in this setting, the mean age of peak helminthiasis prevalence decreases as transmission pressure increases, with non-school children below 18 years old being most at risk of acquiring parasitic infections. This was the first baseline survey of parasitic infections in Milola Ward, so the results will be crucial for guiding control efforts against parasitic diseases in the area.

## INTRODUCTION

Schistosomiasis and soil-transmitted helminthiases are among the major neglected tropical diseases.^1^ Schistosomiasis, caused by parasitic worms of the genus *Schistosoma*, is endemic in 78 countries, most of which are in sub-Saharan Africa.^2^ About 200 million people are estimated to have schistosomiasis, while 800 million are at risk of acquiring the disease.^3,4^ On the other hand, recent estimates indicate that soil-transmitted helminths (STHs) infect even more people, with over 800 million being infected with *Ascaris lumbricoides*, 465 million with *Trichuris trichiura*, and around 400 million with hookworms (*Necator americanus* and *Ancylostoma duodenale*).^5^ Schistosomiasis and soil-transmitted helminthiases occur mostly in the poorest parts of the world, where they have profound negative effects on the welfare and productivity of the affected people. Infection with STHs impairs growth and cognitive development, particularly among children, resulting in poor educational achievements and reduced productivity.^6^

There are 2 forms of schistosomiasis. One of them is uri-nary schistosomiasis (caused by *S. haematobium*), which initially leads to haematuria and can have severe effects on the organs of the urogenital system, including the bladder, urethra, uterus, and vagina.^7^ The other form is intestinal schistosomiasis, which can be caused by any of the other 4 major schistosome species that infect humans, namely, *S. mansoni, S. japonicum, S. menkongi*, and *S. intercalatum*. Intestinal schistosomiasis can cause abdominal pain, diarrhoea, stunted growth, and impaired cognitive abilities in children.^1,2,8,9^ Chronic infections can also damage internal organs, such as the liver, spleen, and gallbladder.^2^

Both *S. mansoni* and *S. haematobium* exist in Tanzania, with marked focal variations in endemic areas.^10–14^ Recent estimates indicate that *S. mansoni* infection is most prevalent around the Lake Victoria basin, while *S. haematobium* infection is distributed along the coast of the Indian Ocean as well as the inland areas and hinterland of Lake Victoria.^14,15^ While *S. mansoni* is more focal and virtually absent in the coastal regions and on the Unguja and Pemba islands, *S. haematobium* is widespread in the country, including on the isles.^15,16^ Estimates in 1977 indicated that 19% of the people in Tanzania were at risk of acquiring schistosomiasis,^11^ while recent reports suggest that by 2010, about 23 million Tanzanians were infected with schistosomiasis, representing an overall prevalence of 53.3%.^17^ Even higher schistosomiasis prevalence rates have been reported in recent years in some areas.^14,18^ Despite that, current and adequate information on the distribution of schistosomiasis and soil-transmitted helminthiases is not available for most of Tanzania, particularly in Lindi District in the south of the country.^15,18^ As a result, information on the burden of schistosomiasis for most areas, including Milola Ward in Lindi District, has been based mainly on hospital reports.^11,15^ Such information is liable to inaccuracy and unreliability due to poor recording and lack of random sampling,^19^ and the data may not be useful for designing effective disease control programmes.^20^ Brooker and colleagues^15^ conducted a countrywide survey on the distribution of schistosomiasis in Tanzania from 1980 to 2009 and indicated that schistosomiasis was most likely endemic to Lindi Region, although no field assessment was made on the local distribution of the disease in the region. Building on that situation, this study applied standard field epidemiological techniques to investigate the current status of schistosomiasis and produce up-to-date data on schistosomiasis at the focal level in Milola Ward, southeastern Tanzania. Information on the health status of Milola Ward, particularly of children and other at-risk sections of the population would help guide development programmes, including educational, community welfare, and livelihood support programmes in the area. The results obtained will enhance the understanding of schistosomiasis and soil-transmitted helminthiases in these areas and contribute useful information for controlling the diseases. Furthermore, for control and prevention of morbidity due to schistosomiasis, the World Health Organization (WHO) recommends regular treatment for at-risk groups with praziquantel.^21^ Since identifying the high-risk populations and establishing treatment frequency both depend on the prevalence of infection,^21,22^ such data are important for guiding treatment and control priorities. This study is also in line with the Tanzanian government's current programme to improve the health and welfare of its people through the control and elimination of infectious diseases, particularly neglected tropical diseases, such as schistosomiasis and soil-transmitted helminthiases.^23^ This was the first baseline survey of parasitic infections in Milola Ward. Thus, the results will be crucial for guiding control efforts for parasitic diseases in the area. The transmission of schistosomiasis and soil-transmitted helminthiases is largely determined by host characteristics, such as sex, age, immunity, and economic status as well as the environmental factors, which include temperature, rainfall, humidity, landscape, and land use patterns.^24^ This study investigated the possible influence of both the host (age and sex) and the environment factors (village location) to obtain a true picture of the patterns of schistosomiasis and soil-transmitted helminthiasis in the study area.

### Objectives

The study's main objective was to produce baseline information about infections caused by schistosomes and STHs (geohelminths) in Milola Ward. The specific objectives were (1) to determine the infection prevalence and intensity of *S. mansoni, S. haematobium*, and STHs among 250 residents of Milola Ward by the end of the study period; (2) to establish the influence of locality on schistosome transmission in Milola Ward by the third quarter of the study period; and (3) to determine the variation of schistosome infection between children and adults, and between males and females in Milola Ward by the third quarter of the study period.

## METHODS

### Study Area

**FIGURE 1.**
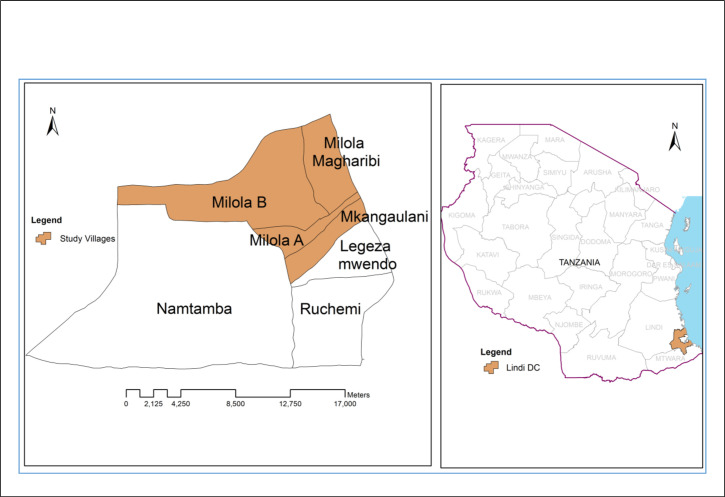
Maps of Tanzania Indicating the 4 Milola Ward Study Villages and the Location of Lindi District in Southeastern Tanzania

This study was conducted in Milola Ward located in Lindi Rural District in Lindi Region, southeastern Tanzania (**Figure 1**). Milola Ward is a rural area inhabited mostly by farmers. The ward was selected for this study because it is among the areas in Tanzania lacking current information on the levels of schistosomiasis and other helminth infections.^14,15,18^ At the time of study initiation, the only control and preventive measures against schistosomiasis and soil-transmitted helminthiases in the study area were the school-based annual mass drug administration (MDA) programmes and the occasional distribution of antihelminthics to adults during government-organised neglected tropical disease campaigns, which this study aimed to promote. Milola Ward is also a focal site for the Michigan State University–Tanzania Partnership Program,^25^ which funded the study. The ward is administratively divided into 7 villages. However, only 4 of them – Milola A, Milola B, Milola West (Magharibi), and Mkanga Ulani – were covered in the present study, while the 3 villages of Namtamba, Legezamwendo, and Ruchemi were left out due to inaccessibility (**Figure 1**). For each included village, all subvillages (*Vitongoji* in Kiswahili) were included in the sampling. A summary of the estimated population sizes for each village, based on the 2012 National Census report, is shown in **Table 1**.

**TABLE 1.** Number of Residents and Study Participants in Milola Ward, as per 2012 Population and Housing Census

Village	Total Residents n	Male Participants n	Female Participants n	Total Participants n
Namtamba^a^	798	–	–	–
Milola A	3,347	19	32	51
Milola B	1,508	14	11	25
Milola West (Magharibi)	976	25	45	70
Mkanga Ulani	1,647	27	22	49
Legezamwendo^a^	729	–	–	–
Ruchemi^a^	612	–	–	–
**Total**	**9,617**^b^	**85**	**110**	**195**

a Not sampled due to inacessibility.

b 9,616 in 2012 census report.

### Study Design

A sample size of 250 participants was taken for the study. This was based on the WHO recommendation that 250 participants are considered sufficient for studies seeking to establish baseline data on the prevalence and intensity of schistosomiasis and soil-transmitted helminthiasis in a homogeneous geographical area.^24^ A homogeneous area in this context is defined as an area with similar climate, humidity, ecology, and soil conditions^24^ (**Figure 1**). This sample size allows for comparison of results with other studies, which would be useful for assessing the success of control programmes.^24^ Although about 63 participants would be sampled from each village to make the recommended sample size of 250, we enrolled a higher number of participants (n=85) from each village, assuming around a 30% to 40% dropout rate due to attrition or non-compliance.^26^ Systematic sampling was applied by selecting every third household in each studied village. A set of 2 stool samples and 2 urine samples was requested from each participant. This study was only conducted during the dry season due to limited resources. Participants testing positive for schistosomiasis or a soil-transmitted helminthiasis were referred to Milola Health Centre for treatment.

### Inclusion and Exclusion Criteria

Participants were included and excluded based on criteria published elsewhere.^27,28^ All residents of each selected household were sampled except school children, who were excluded because they had just been treated with praziquantel and antihelminthic drugs as part of the Tanzanian government's programme against schistosomiasis and soil-transmitted helminthiases in primary schools. Only people aged between 5 and 90 years who were presumed to be active enough to be at a reasonable risk of acquiring parasites were eligible to be included in the study. Children aged less than 5 years were deemed to be at lower risk of contracting schistosomiasis and geohelminthiases and were excluded from the study, although infant infections have been documented.^29^ Adults over 90 years and seriously ill people who would not be physically able to participate in the study were also excluded. Other excluded groups included pregnant women and new residents of the study area to avoid infections imported from elsewhere. Moreover, to avoid reporting false prevalence data, adults and non-school children treated with praziquantel or albendazole within the previous month were also excluded from the study. Participants were asked about this information during the enrolment exercise and responses were entered into a questionnaire.

Only participants granting informed consent (oral or written) were enrolled in the study. On the sampling day, participants were counselled on the goals of the study and the implications of their participation. Full information was provided to the participants on the study's benefits and risks in Kiswahili, the language widely spoken in the area. It was explained to them clearly that they were free not to participate in the study and that they could withdraw from it at any time without seeking permission. Full consent was sought, and those agreeing to participate were asked to sign a consent form or indicate consent orally. For minors (children), consent was sought from their parent or guardians, and in situations where a child refused participation, he or she was excluded from the study.

### Sample Collection and Examination

**Photo 1.**
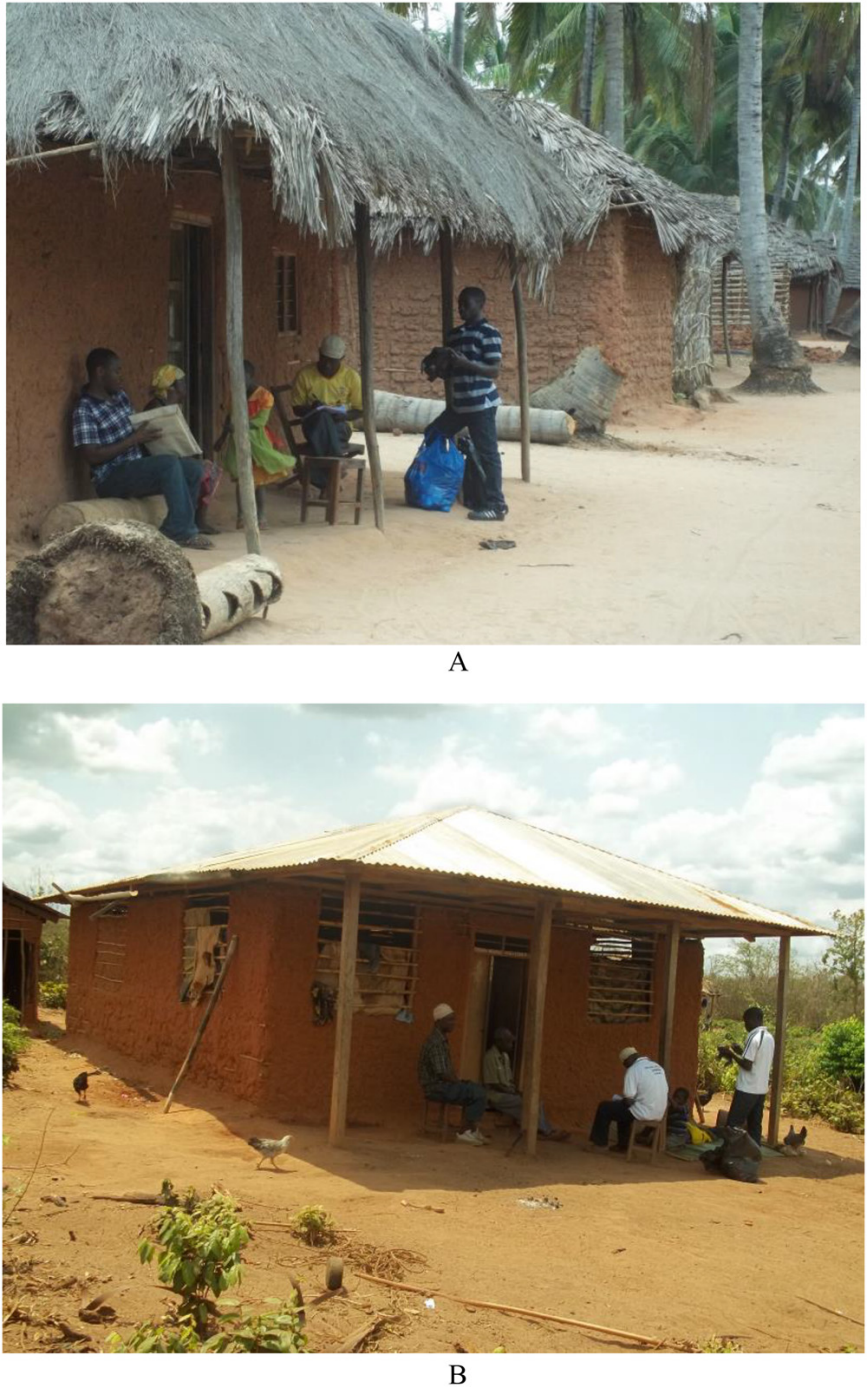
Sampling materials were distributed to each participant at their home (A & B) at Milola Ward in southeastern Tanzania, and instructions were given on the protocols for stool and urine collection

**Photo 2.**
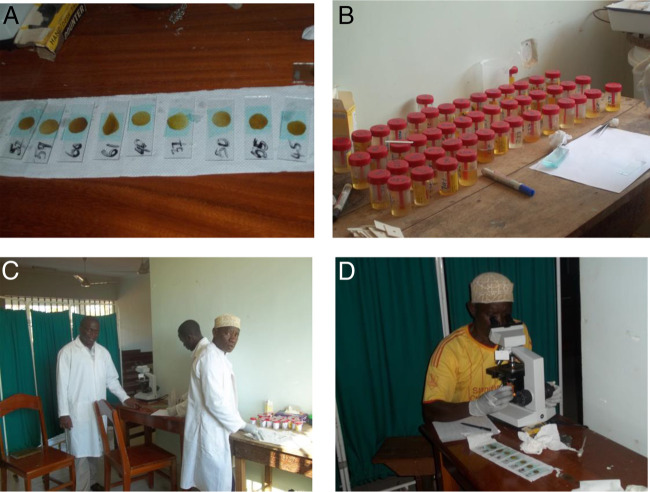
Kato–Katz slides smeared with faecal samples (A) and plastic vials containing urine samples (B) being processed and analysed in the laboratory at Milola Health Centre, Lindi District, in southeastern Tanzania. Members of the research team in action in the laboratory (C & D), including the author (standing between chairs in C)

Sample collection and examination was conducted from September to October 2014. Sampling materials were distributed to each participant, and instructions about stool and urine collection procedures were provided (**Photo 1**). The materials included a wooden spatula for picking up stool, 2 plastic vials (120 ml) for depositing stool and urine, respectively, and a polythene bag for storing the vials. The vials for depositing stool and urine samples were labelled with the participant's name, sex, village, and subvillage, as well as the collection date. The materials were distributed on the morning of the first day and collected by members of the study team on the morning of the following day. Two sets of sampling materials were distributed to each participant on 2 days separated by a 7-day interval. Participants were instructed to collect urine between 10:00 and 14:00, as these are peak times for *S. haematobium* excretion.^30^ The processing and examination of stool and urine samples were conducted at Milola Health Centre in Milola A village (**Photo 2**). Stool was examined for *S. mansoni* and STHs using the Kato–Katz technique, and urine was examined for *S. haematobium* using the filtration technique; these 2 methods are widely recommended for this kind of study.^30^ All observed parasites were identified and counted using standard guidelines and procedures for parasite recovery and identification based on morphology, size, and shape of eggs and larvae.^28,31^ We did not specify egg counts above 50 eggs per 10 ml of urine during examination because we were mostly interested in the categorical identification of infection intensities (low, medium, and high), and so intensities were capped at 50 eggs per 10 ml of urine. Participants with more than 50 *S. haematobium* eggs per 10 ml of urine were categorised as heavily infected, as per WHO guidelines.^32^

### Data Analysis

The terms “prevalence”, “intensity” (egg count), and “mean intensity” were used as indicators of parasitic infection, and we applied appropriate analyses recommended for studies on parasite ecology.^24,30^ Infection prevalence was calculated as the percentage of infected individuals out of all examined individuals, and intensity was the number of *S. haematobium* eggs per 10 ml of urine. Although we targeted a set of 2 stool and urine samples, most participants did not bring the second sample. The analysis was thus based on a single urine and single faecal sample from each participant. To obtain the number of eggs per gram (epg), which is the standard measurement of the intensity of infection at the individual level, the number of faecal helminth eggs (counted on the slide using the Kato–Katz technique) was multiplied by an appropriate number (multiplication factor), which depends on the size of the template hole used.^30,32^ The template hole used in the present study could hold 41.7 mg of faeces, so the number of faecal eggs per slide was multiplied by 24. The mean epg was then used as a proxy of worm burden.^32^ Worm burden for *S. haematobium* infection for each participant was estimated as the number of eggs per 10 ml of urine.^33^ Levels of STH and *S. haematobium* infection (intensity) were categorised as “heavy”, “moderate”, and “light” infections, respectively, as per WHO guidelines.^32^ Normality testing of the samples indicated that parasite egg counts (intensity) strongly deviated from the normal distribution, as variance was larger than the mean. Non-parametric tests were therefore applied, with the Mann–Whitney U-test used to analyse the variation of parasite intensity between male and female participants and between age groups (children and adults).^34^ Linear regression analysis was used to measure the association of age with parasite intensity, and the Kruskal–Wallis test was applied to determine the variation of parasite intensity among villages and test whether the proportion of infected individuals varied significantly among localities.^35^ The Pearson chi-square (X^2^) test of independence was used to analyse for variation of *S. haematobium* prevalence between the strata of categorical variables. Analyses were performed using Stata, version 11 (StataCorp LLC, College Station, TX, USA), and the statistical significance level was *P*<.05.

### Ethical Considerations

Ethical approval for this study (Ref. No. NIMR/HQ/R.8a/Vol. IX/1798) was provided by the National Institute for Medical Research, Tanzania.

## RESULTS

A total of 195 participants returned faecal and urine samples (**Table 1**). However, only 190 (97%) participants submitted viable and analysable urine samples, including 107 (56%) females and 83 (44%) males (**Table 2**). On the other hand, only 158 (81%) of 195 participants supplied faecal samples in sufficient condition for parasite detection, 37 (23%) of whom were children (**Table 2**). The types of parasites observed were *S. haematobium*, which was diagnosed from urine samples, as well as *T. trichiura* and hookworms obtained from stool. *S. haematobium* was found in 44 (23%) of 190 urine samples, followed by hookworms diagnosed in 12 (8%) of 158 examined stool samples, and *T. trichiura* observed in 6 (4%) of 158 stool samples (**Table 3**).

**TABLE 2.** Prevalence of Urinary Schistosomiasis by Village, Sex, and Age Group Among Milola Ward Residents

Variable or Category	Participants Examined n	Participants Positive n	Prevalence %	*P* Value
**Village**				
Milola A	51	10	20	
Milola B	23	6	26	
Milola West	69	17	25	.88
Mkanga Ulani	47	11	23	
Total	190	44	23	
**Sex**				
Female	107	27	25	
Male	83	17	20	.47
Total	190	44	23	
**Age**				
Child	37	18	49	
Adult	153	26	17	<.001
Total	190	44	23	

**TABLE 3.** Overall Prevalence and Intensity of Parasite Types Identified from Study Participants

Parasite Species	Sample Type	Participants Examined n	Participants Infected n	Prevalence %	Intensity
Hookworms	stool	158	12	8	21.4 eggs/Kato–Katz slide
*Trichuris trichiura*	stool	158	6	4	0.72 eggs/Kato–Katz slide
*Schistosoma haematobium*	urine	190	44	23	5.2 eggs/10 ml urine

The prevalence and intensity of *S. haematobium* did not vary significantly between villages (intensity, X^2^=0.4 with 3 degrees of freedom [df], *P*=.95; prevalence, X^2^=0.7; *P*=.88) (**Table 2** and **Table 4**). There were 6 participants, 2 each from the villages of Mkanga Ulani, Milola B, and Milola West, who had heavy *S. haematobium* infections. As shown in **Table 2**, the prevalence of *S. haematobium* infection was higher among the 107 female participants (n=27, 25%) than among the 83 males (n=17, 20%). However, this variation was not statistically significant (X^2^=0.5; *P*=.47) (**Table 2**). Similarly, variation of the intensity of *S. haematobium* in females (5.21 epg) and males (5.11 epg) was not statistically significant (X^2^=0.2 with 1 df; *P*=.66), as also shown in the odds ratio analysis results in **Table 4**.

**TABLE 4.** Odds Ratio Analysis Results Showing Significantly Higher Intensity of *S. haematobium* in Children Compared to Adults (*P*<.001)

	Crude OR		Adjusted OR	
Variable	OR (95% CI)	*P* Value	OR (95% CI)	*P* Value
**Village**				
Molola A	1.0	–	1.0	–
Molola B	1.25 (0.48–3.29)	.65	1.33 (0.48–3.71)	.59
Mkanga Ulani	1.34 (0.55–3.24)	.52	1.34 (0.53–3.38)	.54
Milola West	1.54 (0.48–4.93)	.47	1.63 (0.47–5.68)	.44
**Sex**				
Male	1.0	–	1.0	–
Female	1.29 (0.65–2.57)	.47	1.72 (0.80–3.70)	.17
**Age**				
Adult	1.0	–	–	–
Child	4.59 (2.12–9.92)	<.001	5.09 (2.29–11.32)	<.001

Abbreviations: CI, confidence interval; OR, odds ratio.

**FIGURE 2.**
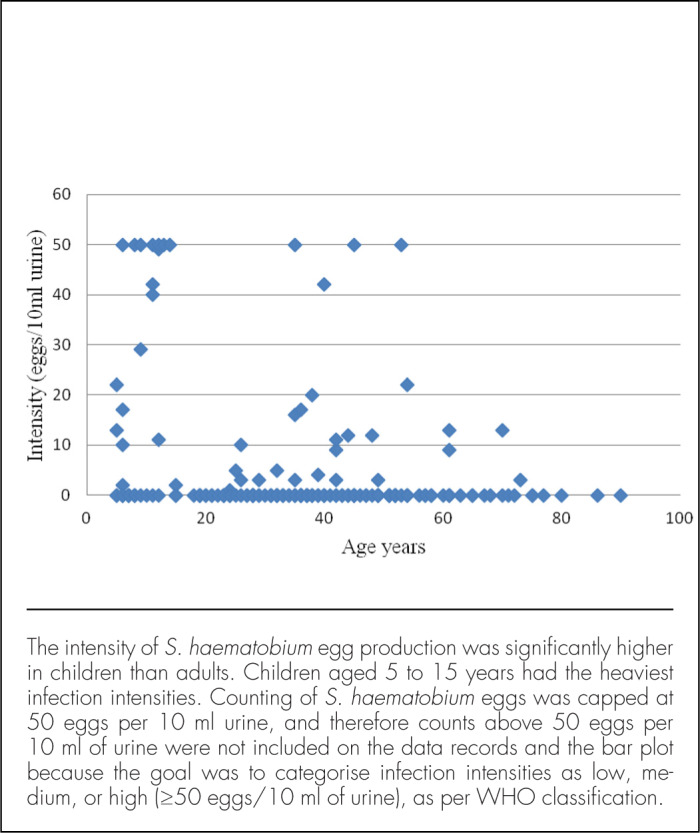
Relationship Between *Schistosoma haematobium* Direct Egg Counts and Participant Age as a Continuous Variable

**FIGURE 3.**
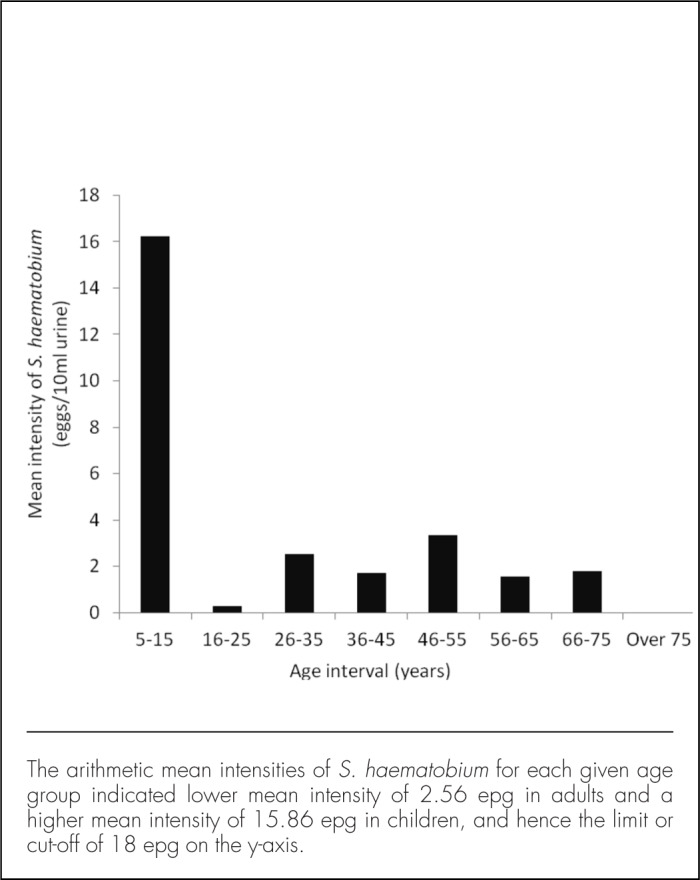
Variation in *Schistosoma haematobium* Mean Intensity With Participant Age, indicated by the Arithmetic Mean Intensities for Given Age Intervals.

Thirty-seven children were sampled during the study, ranging from 5 to 15 years of age, with the majority aged 6 to 8 years (**Table 5**). The 121 adults sampled for stool were between 18 and 90 years of age, without a clear predominance of any particular age group (**Table 5**). The prevalence of *S. haematobium* was higher among the 37 children (n=18, 49%) than the 153 adults (n=26, 17%), and the difference was statistically significant (X^2^=16.6; *P*<.001) (**Table 2**). The linearity of the relationship between the intensity of *S. haematobium* infection and human age is demonstrated in **Figure 2** (with egg counts capped at 50 eggs per 10 ml of urine). Linear regression analysis of *S. haematobium* egg intensity (dependent variable) with participants' age intervals revealed that mean egg counts were higher in children (15.86 epg) compared to adults (2.56 epg) (Figure 3), and this difference was statistically significant (*P*<.001) (**Table 4**).

**TABLE 5.** Age Distribution of Non-School Children and Adults Who Participated in the Present Study at Milola Ward in Lindi District, Tanzania (N=195)

Age (Years)	n
**Children**	
5	4
6	8
7	4
8	4
9	3
10	1
11	4
12	4
13	1
14	1
15	3
Total	37
**Adults**	
18–22	10
23–27	15
28–32	23
33–37	27
38–42	17
43–47	13
48–52	18
53–57	9
58–62	8
63–67	4
68–72	5
73–77	4
Over 77	5
Unknown	1^a^
Total	158

a Unknown age: excluded in data analysis.

The intensity of *S. haematobium* egg production was significantly higher in children than adults. Children aged 5 to 15 years had the heaviest infection intensities. Counting of *S. haematobium* eggs was capped at 50 eggs per 10 ml urine, and therefore counts above 50 eggs per 10 ml of urine were not included on the data records and the bar plot because the goal was to categorise infection intensities as low, medium, or high (≥50 eggs/10 ml of urine), as per WHO classification.

The arithmetic mean intensities of *S. haematobium* for each given age group indicated lower mean intensity of 2.56 epg in adults and a higher mean intensity of 15.86 epg in children, and hence the limit or cut-off of 18 epg on the y-axis.

### Availability of Data and Materials

The datasets supporting the conclusions of this article are available. Contact the corresponding author for permission and access to the datasets.

## DISCUSSION

### Observed Levels of Parasitic Infection

The parasite burden observed among study participants in Milola Ward in southeastern Tanzania has significant public health implications for this area. For instance, hookworms and whipworms (*T. Trichiura*) are major STHs that cause significant health impairments, including iron-deficiency anaemia, malnutrition, and growth retardation.^24^ Another parasite diagnosed was *S. haematobium*, which is a blood fluke that causes urinary schistosomiasis, a potentially debilitating disease, the chronic form of which can lead to kidney failure and bladder cancer if untreated.^36^ The drugs used for these infections – albendazole for hookworm disease and trichuriasis and praziquantel for schistosomiasis – should, therefore, be made available regularly at Milola Ward Health Centre. The drugs should be offered free of charge or highly subsidised because, otherwise, most people in the villages would not be able to afford them.

Despite being relatively small in both scope and duration, our study demonstrated prevalence levels, particularly for *S. haematobium*, that the Tanzanian government could use in planning control programmes according to WHO guidelines, which specify prevalence-based treatment frequency.^37,38^ We observed that although most infected participants (97%) had a mean prevalence of *S. haematobium* within the light or low infection level (1 to 49 eggs per 10 ml of urine), 3% of them (not included in the analysis) had more than 50 eggs per 10 ml of urine, which is categorised by WHO as heavy infection.^32^ The government could, therefore, consider adjusting the frequency of treatment in these areas. According to available records, the current school-based MDA programmes are conducted once each year, which does not match our observed prevalence levels. Over 20% prevalence of *S. haematobium* was found in some areas (**Table 2**). We recommend that the frequency of treatment be increased to twice per year, in accordance with the WHO recommended frequency when prevalence is above 20%.^38^

### The Influence of Age on Parasite Infections in Milola Ward

Although non-school children sampled in the present study were far fewer than adults, the former had heavier parasite burdens. Overall, more children were infected with hook-worms and *S. haematobium* parasites than adults, and peak parasite intensities and prevalence rates were found in children aged between 5 and 15 years (**Figure 2**). These findings confirm the widely known relationship between prevalence and age, wherein the mean age of peak prevalence in a population decreases as transmission pressure increases (**Figure 2** and **Figure 3**).^39,40^ The present results are consistent with reports by other researchers who have also reported a slow rise in schistosomiasis prevalence and intensity in children followed by an equally gradual decline in adults.^36,41^ Children are more susceptible to infections compared to adults largely because they lack acquired immunity and engage in behaviours and activities that bring them in contact with infested water or soil.^41–43^ Most children in Milola Ward were seen walking and playing barefoot at the time of the study, and it is possible that they got infected with STHs, such as hookworms and whipworms (*Trichuris*), through contact with the soil. In the local context, however, the results affirm that age influences parasite transmission in Milola Ward, where children below 18 years old are at higher risk of acquiring the infections compared to adults. Other studies, in similar and different settings, have demonstrated the association between participant age and schistosomiasis, with a gradual increase in both prevalence and intensity in children, followed by a slow decline in adults.^41,42,44^

Just before the start of the present study, the Tanzanian government was conducting its regular school-based distribution of antihelminthic drugs to children in the study area. Thus, to avoid reporting false prevalence, and in cognizance of the suggestion that post-MDA sampling for schistosomiasis should be conducted at least 6 months after drug delivery,^42^ school children were excluded from this study. The studied groups (adults and non-school children) had not been involved in the government-sponsored MDA programmes. Most (25 of 37) of the non-school children included in this study were 7 years of age or older (**Table 5**), which is the school age in Tanzania. For unknown reasons, these children were not attending school at the time of the study. In school, they would likely have received praziquantel and albendazole during the MDA campaigns. The disproportionately higher prevalence of schistosome and hookworm infection among non-school children in Milola Ward could, therefore, be due to missed opportunities for school-aged children to benefit from the government's National Schistosomiasis Control Programme. The current school-based treatment programme, which targets school children only, should also include non-school children, as they were heavily infected with schistosomiasis but did not have regular access to drugs. Further investigations should also be conducted on the strengths and weaknesses of school- and community-based treatment approaches to determine which is better for specific areas. These findings could also form a useful basis for the planning and implementation of control measures against parasitic diseases in these areas. Future studies comparing the levels of parasitic infection between school and non-school children in the area and other similar settings would enable us to better understand the factors influencing the levels of schistosomiasis and STHs between the 2 groups and take appropriate actions. Furthermore, the causes of differences in the acquisition and progression of these infections in children and adults in Milola Ward can be fully understood if comprehensive surveillance is conducted that encompasses the wet and dry seasons, as well as a broader range of environmental and socioeconomic factors.

### Limitations

Compliance to sampling in the present study was not consistent among participants. For instance, in the initial sampling exercise, 158 participants brought back faecal samples but, for whatever reasons, only 26 participants provided the second stool sample. Similarly, for urine sampling, 190 participants provided a first urine sample, but only 53 provided a second specimen. It is also possible that some adults consciously decided not to provide samples. As such, our analysis and discussion have focused only on data from the initial sampling, which may have reduced the chances of a broader interpretation of the findings. As noted elsewhere,^24^ epidemiological sampling of adults can be challenging and difficult. Thus, school children are an ideal target group for sampling, especially in resource-constrained settings, to allow for more representative and reliable results.

## RECOMMENDATIONS

The present study has not established the transmission points or foci for schistosome infection in the sampled villages. To that end, the sources of schistosome infection remain unknown. Future studies should, therefore, aim to establish the risk areas, such as specific water bodies and farms, and determine how human movements to and away from these areas expose them to the risk of infection. Since snails are critical for the lifecycle and transmission of schistosomes, a comprehensive survey of snails in Milola River and other local water bodies is essential for a full understanding of the transmission patterns and dynamics of urinary schistosomiasis in the area. Although there are already some studies, at least at the national level in Tanzania, that have used geographic information system (GIS) mapping to model the transmission risk levels for soil-transmitted helminthiases and schistosomiasis across the country, based on known climatic, soil, and other environmental variables,^15,45^ such studies are lacking at the district and community levels. As such, the use of GIS for mapping and identifying risk areas (transmission hotspots) would help to resolve the questions of how and where the people at Milola Ward acquire urinary schistosomiasis and soil-transmitted helminthiases. Land usage patterns in Lindi District and their impact on schistosomiasis and soil-transmitted helminthiasis distribution should also be analysed to identify priority areas for interventions, to estimate intervention needs and to assess the progress of control programmes against these infections.

## CONCLUSION

This study focused on the demographic factors driving schistosomiasis and soil-transmitted helminthiases at Milola Ward in Lindi District, Tanzania. The major parasites present in the population were *S. haematobium, T. trichiura*, and hookworms. Across the studied villages, age was the most important factor influencing parasite transmission. The chances of acquiring schistosomiasis and hookworm disease in the area were higher among non-school children than among adults, most likely due to the former's susceptibility to parasitic infections. It is possible that non-school children involved in the present study had infection levels that were higher than expected because they did not receive the school-based treatment. At the time of the study, adults were also not receiving regular treatment and were not covered by an MDA programme. The present study provides baseline information on the distribution of schistosomiasis and soil-transmitted helminthiases in the area, which can be used as guidance for rolling out control and intervention efforts. Because the study was conducted only during the dry season, it is possible that the levels of parasitic infections were underestimated, as the rainy season is more conducive for schistosome and STH transmission. More comprehensive future epidemiological studies in Milola Ward may provide more insights on the patterns and dynamics of parasitic infections in the area.
